# Context-Dependent Roles of Siglec-F^+^ Neutrophils

**DOI:** 10.3390/biomedicines13112601

**Published:** 2025-10-24

**Authors:** Kisung Sheen, Taesoo Choi, Man S. Kim

**Affiliations:** 1 Translational-Transdisciplinary Research Center, Medical Science Research Institute, Kyung Hee University Hospital at Gangdong, Kyung Hee University College of Medicine, Seoul 05278, Republic of Korea; kisungsheen@khu.ac.kr; 2 Division of Urology, School of Medicine, Kyung Hee University, Seoul 05278, Republic of Korea; taesoochoi85@hanmail.net; 3 Department of Biomedical Science and Technology, Graduate School, Kyung Hee University, Seoul 05278, Republic of Korea

**Keywords:** neutrophil heterogeneity, Siglec-F, Siglec-8, NETosis, ROS, anti-apoptosis, long-lived neutrophil, eosinophil

## Abstract

Recent studies in murine disease models have identified Siglec-F^+^ neutrophils, which express a marker traditionally associated with eosinophils, as a functionally distinct population characterized by extended lifespans and context-dependent roles. While conventional neutrophils typically return to the bone marrow or undergo apoptosis at the site of inflammation, these cells remain in tissues for extended periods. These cells demonstrate remarkable functional plasticity, promote bacterial clearance and immune activation during infections, foster immunosuppression and tumor progression in cancer, and contribute to tissue remodeling in fibrotic diseases. In this review, we examine the key features governing Siglec-F^+^ neutrophil differentiation and function—including Siglec-F signaling, metabolic programming, and upstream cytokine cues—and explore how targeting these pathways may offer promising avenues for precision immunomodulation.

## 1. Introduction

Neutrophils, the most abundant circulating leukocytes, have long been considered the homogeneous first responders of innate immunity. This traditional view has been challenged by recent discoveries of their remarkable heterogeneity, with specialized subsets exhibiting distinct functional properties [1,2,3,4,5,6,7,8,9,10]. Among these, neutrophils expressing Siglec-F have emerged as a key population, identified across a wide spectrum of murine pathologies, from various cancers [11,12,13,14,15,16,17,18,19,20,21,22], to non-cancer contexts such as inflammation [23,24,25,26,27,28,29,30], bacterial infection [31,32,33], infarction [34,35], fibrosis [36,37] and even steady state [38].

While the generation of these cells often involves tissue-specific reprogramming of conventional neutrophils, the driving signals are remarkably diverse and context-dependent. These range from canonical cytokines (G-CSF or GM-CSF) [18,27,29,36,37], microbial or environmental stimuli (LPS, extracellular ATP), and systemic of metabolic factors such as osteoblast-derived signals in lung cancer [11] or specific lipids in MASH-HCC [19]. Similarly, while features such as an extended lifespan and enhanced effector functions are observed in several cases, their ultimate phenotype is dictated by the specific tissue microenvironment, resulting in a functionally plastic population [11,12,13,14,15,16,17,18,19,20,21,22,23,24,25,26,27,28,29,30,31,32,33,34,35,36,37,38].

Most critically, these cells display a striking functional dichotomy. Their role is highly context-dependent: in the upper airways, they provide protective antimicrobial defense, whereas in the lungs, heart, and kidneys, their potent effector functions drive pathological inflammation and fibrotic tissue remodeling [25,26,27,28,29,30,33,34,35,36,37]. In cancer, they predominantly promote tumor progression through profound immunosuppression [11,12,13,14,15,16,18,19,20,21,22].

Despite their clear importance in preclinical models, a critical translational gap exists, as the relationship between murine Siglec-F^+^ neutrophils and their potential human counterpart, Siglec-8^+^ neutrophils, remains largely unexplored. This review comprehensively examines the mechanisms governing Siglec-F^+^ neutrophil generation and their diverse, context-dependent functions. We further analyze the molecular basis of Siglec-F signaling and discuss emerging therapeutic strategies that aim to precisely modulate these plastic and potent immune cells.

## 2. Mechanisms of Siglec-F^+^ Neutrophil Generation

### 2.1. Normal Neutrophil Development and Tissue-Specific Conversion

Neutrophils develop in the bone marrow through granulopoiesis, a process driven by G-CSF, which controls their proliferation, differentiation, and mobilization [37,38,39,40,41]. Under homeostatic conditions, their release is controlled by a balance between CXCR4-mediated retention and CXCR2-mediated egress signals [41]. G-CSF disrupts this equilibrium by downregulating CXCR4, allowing only mature, segmented neutrophils to enter the circulation, where they have a brief half-life of 6–24 h [29,30,31,32,33,34,35,36,37,38,39,40,41,42,43].

This process is altered during severe inflammation, leading to “emergency granulopoiesis” and the accelerated release of both mature and immature neutrophil precursors [43]. This premature release of immature forms is clinically known as a “left-shift,” a term referring to their appearance on the left side of the traditional maturation spectrum [41,42,43]. These immature cells are phenotypically distinct (e.g., CD16^dim^ CD62L^bright^ and CD10^−^ in humans) and have reduced antimicrobial capacity compared to their mature counterparts [5,7].

Contrary to the traditional view of neutrophils as terminally differentiated, it is now recognized that they retain significant transcriptional plasticity after entering tissues. Neutrophils retain the machinery for inducible protein synthesis post-differentiation; their total mRNA content is 10- to 20-fold lower than that of other leukocytes [5]. This retained capacity is the key mechanism that allows them to adapt to local microenvironments by undergoing further differentiation in response to specific tissue cues.

This plasticity is the basis for the well-documented heterogeneity of neutrophils in peripheral tissues. One of the latest examples of this phenomenon is the population of Siglec-F^+^ neutrophils. First characterized in a preclinical lung adenocarcinoma model, this subset emerges when conventional neutrophils acquire the eosinophil-associated marker Siglec-F after tissue infiltration [11]. This conversion is driven by local factors, particularly GM-CSF and TGF-β1, and results in a population with an extended lifespan and novel functions, such as the ability to produce collagen [34]. The study of these cells therefore provides a clear model for how local cues shape neutrophil identity and function in disease.

### 2.2. Existing Neutrophil Classification Systems and the Position of Siglec-F^+^ Subsets

The traditional view of neutrophils as a homogeneous population has been replaced by the recognition of their remarkable heterogeneity [5,6,7,8,9,10]. However, as some have critically noted, current functional concepts often rely on simplified paradigms that may not capture the full complexity of neutrophil biology in disease. Three major classification systems have emerged: (1) Functional polarization (N1/N2), which categorizes tumor-associated neutrophils (TANs) as either anti-tumoral (N1) or pro-tumoral (N2) based on their inflammatory state [7]; (2) immunosuppressive function (PMN-MDSCs), which defines polymorphonuclear myeloid-derived suppressor cells by their potent T cell suppression [8,9,18]; and (3) physical density (HDNs/LDNs), which separates mature, high-density neutrophils (HDNs) from activated or immature low-density neutrophils (LDNs) [10]. A significant challenge in the field is that these definitions often describe overlapping cell populations, suggesting they may represent different experimental views of the same biological states rather than truly distinct lineages [7].

Within this complex landscape, Siglec-F^+^ neutrophils have emerged as a population that challenges straightforward classification. We propose that Siglec-F expression does not define an entirely new lineage but rather serves as a state-dependent marker that identifies terminally differentiated neutrophils that have undergone potent, tissue-specific programming. This interpretation aligns with recent calls to move beyond marker-based classifications and toward a more functional understanding to facilitate the transition from experimental insight to clinical translation.

This hypothesis is strongly supported by recent single-cell RNA sequencing data. A study by Veglia et al. identified that the most immunosuppressive subset of PMN-MDSCs (designated PMN3) was uniquely characterized by high co-expression of CD14 and Siglec-F [18]. These CD14^high^Siglec-F^+^ cells exhibited the highest levels of suppressive molecules (Arg1, Nos2, PD-L1) and the strongest T cell suppression capacity. Notably, PD-L1 expression has been reported in various tumor-associated neutrophil subsets [6,44,45]. This finding suggests that Siglec-F may be a superior marker for identifying highly functional effector neutrophils that have been maximally programmed by microenvironmental signals.

This view is further substantiated by several key observations across various disease models. Studies have shown that Siglec-F^+^ neutrophils: (1) mostly arise from conventional neutrophils through tissue-specific reprogramming, not distinct developmental pathways; (2) display remarkable functional plasticity, being protective [23,31] in some contexts but pathogenic in others; (3) exhibit context-dependent survival characteristics [3,8,13]; and (4) comprise multiple transcriptionally distinct subsets even within a single disease [12].

Ultimately, the precise relationship between Siglec-F^+^ neutrophils and existing classifications remains unresolved. Whether Siglec-F^+^ neutrophils represent a functionally distinct population or a common endpoint of various activation pathways requires systematic investigation that moves beyond the current simplified paradigms.

### 2.3. Molecular Mechanisms of Siglec-F Signaling

Siglec-F is a well-established pro-apoptotic receptor on eosinophils that, upon ligand engagement, induces caspase-dependent cell death [46]. In stark contrast, its expression on neutrophils confers a paradoxical pro-survival effect. Siglec-F^+^ neutrophils are often described as mature, long-lived cells, a phenotype supported by evidence of an extended lifespan [13,15,17,36,37], enhanced survival gene programs [36], and resistance to apoptosis [11,13,21].

Structurally, Siglec-F is a CD33-related siglec that recognizes sialic acid ligands via its N-terminal domain. Its cytoplasmic tail contains ITIMs that, upon phosphorylation, recruit the phosphatases SHP-1 and SHP-2 to initiate inhibitory signaling. In eosinophils, this cascade culminates in apoptosis. However, in neutrophils, the downstream consequences of ITIM engagement diverge significantly, suggesting the involvement of cell-specific signaling adaptors.

Recent studies point to the PI3K-AKT pathway as a key driver of this pro-survival phenotype. Tumor-derived G-CSF and GM-CSF prolong neutrophil survival by activating this pathway to upregulate the anti-apoptotic protein Mcl-1 [21]. Additionally, the transcription factor PPARγ has been identified as a key regulator in Siglec-F^+^ neutrophils, potentially linking their survival to metabolic reprogramming [36]. While the direct connection between Siglec-F ligation and PI3K-AKT activation remains to be formally proven, these findings establish a strong mechanistic framework.

This functional dichotomy between cell types likely reflects several factors. Hypotheses include: (1) crosstalk between Siglec-F and growth factor receptor signaling that amplifies PI3K-AKT activation in neutrophils; (2) differences in SHP-1/2 phosphatase substrates between the two cell types; (3) variations in Siglec-F ligands within different tissue microenvironments that trigger distinct signaling outcomes; and (4) a connection between Siglec-F signaling and the metabolic reprogramming that supports extended cell survival [20,21,36].

Understanding these pro-survival mechanisms has significant therapeutic implications, as Siglec-F^+^ neutrophils are associated with poor clinical outcomes. Future studies must identify neutrophil-specific Siglec-F ligands and interacting proteins to develop strategies that can selectively modulate this population without disrupting the homeostatic function of eosinophils. Ultimately, Siglec-F appears to be a contextual modulator of cell fate, integrating glycan recognition with intracellular survival pathways in a manner unique to each cell type.

## 3. Siglec-F^+^ Neutrophils in Noncancerous Diseases: Organ-Specific Manifestations

**Figure 1:**
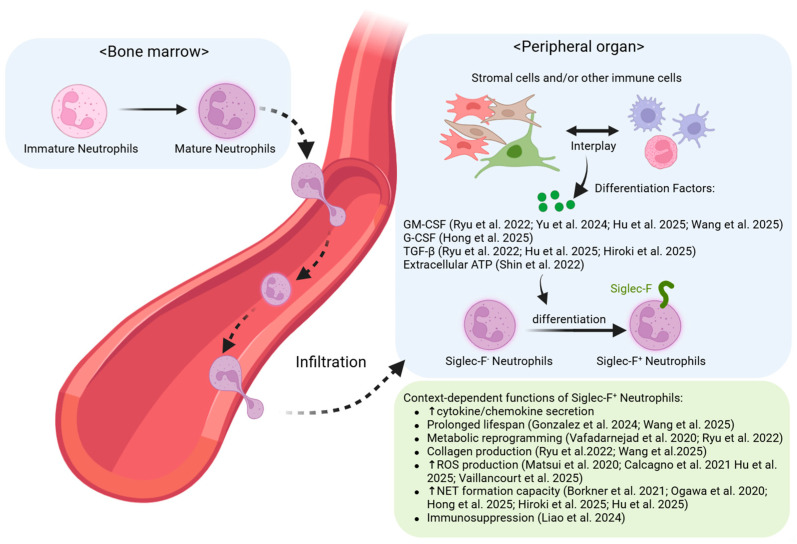
Generation and Functions of Siglec-F^+^ Neutrophils in Non-Cancer Conditions: Siglec-F^+^ neutrophils are generated via tissue-specific differentiation in inflamed or fibrotic peripheral organs. These cells exhibit diverse functions that are either protective (e.g., antimicrobial, tissue repair) or pathogenic (e.g., fibrosis, tissue destruction), depending on the tissue environment. Upward (↑) and downward (↓) arrows denote an increase and a decrease in phenotype or ex-pression, respectively [23,24,25,27,28,29,30,31,32,33,34,35,36,37,38].

In noncancerous diseases, Siglec-F^+^ neutrophils typically arise from bone marrow–derived conventional neutrophils that traffic to peripheral organs and subsequently differentiate in response to tissue-specific stimuli. This overall mechanism is depicted in Figure 1, and the key findings for each organ are summarized in Table 1.

### 3.1. Nasal Cavity: Protective Surveillance and Homeostasis

Within the nasal mucosa, Siglec-F^+^ neutrophils exhibit diverse context-dependent functions, encompassing immune surveillance, host defense, and tissue repair, as delineated by four independent studies.

Under steady-state conditions, Gonzalez et al. identified a long-lived, resident subset (nN3: CD11b^high^ Ly6G^high^ CD11c^+^ Siglec-F^+^) that exists in pathogen-free mice, independent of microbial stimuli [38]. These cells are localized within the subepithelial compartment of the nasal mucosa and are absent from the bone marrow, spleen, and peripheral blood, indicating that they represent a tissue-specific population. They arise from circulating neutrophils through a 5–6 day local differentiation process within the nasal mucosal microenvironment. This transition involves profound transcriptional reprogramming, in which classical neutrophil effector genes (Lysozyme, Lactoferrin, Lipocalin, Calprotectin, TLRs, FPR1/2) are downregulated, while genes associated with antigen processing and presentation are upregulated. Functionally, these cells acquire a unique APC-like phenotype, capable of cross-presenting exogenous antigens to CD8^+^ T cells while retaining their intrinsic phagocytic capacity in vitro. Morphologically, they exhibit hyper-segmented nuclei and dendritic-like processes, consistent with their acquired antigen-presenting functions. Notably, the extended differentiation process lasting up to six days suggests a lifespan inconsistent with the conventional notion of neutrophils as short-lived cells.

During Bordetella pertussis infection, a distinct population of tissue-resident Siglec-F^+^ neutrophils is mobilized by IL-17A to mediate protective immunity in the nasal mucosa [31]. These cells are almost entirely CD45iv^−^ tissue residents and are recruited in an IL-17–dependent manner through the chemokine CXCL1, whose expression increases upon IL-17 stimulation. Siglec-F^+^ neutrophils exhibit higher NETosis than their Siglec-F^−^ counterparts, and their depletion or absence in Il17A^−/−^ mice results in impaired bacterial clearance. This antimicrobial role is further strengthened by stimulation with heat-killed B. pertussis or PMA, which increases NET formation and the expression of antimicrobial peptides such as S100A8 and LCN2. Collectively, these findings identify Siglec-F^+^ neutrophils as key effectors of IL-17–driven, antibody-independent protection in the upper airway [31].

In models of sterile inflammation and repair, Siglec-F^+^ neutrophils also play specialized roles. In the nasal mucosa during allergic rhinitis, Matsui et al. identified this subset as morphologically distinct cells with “botryoid” nuclei, exhibiting enhanced phagocytosis and increased ROS generation, although their precise immunological function remains unclear [24]. More detailed work by Ogawa et al. characterized Ly6G^+^ Siglec-F^+^ double-positive cells (DPCs) in the olfactory neuroepithelium [23]. Using parabiosis and adoptive transfer, they provided direct evidence that these cells are bone marrow-derived and convert locally. The study traced this conversion during acute LPS-induced inflammation and observed their accumulation during the tissue-repair phase of a methimazole-induced injury model. These DPCs, localized exclusively in the olfactory neuroepithelium, retained neutrophil features such as NETosis capacity while gaining neurosupportive potential, expressing neurogenesis-related genes (*Efna5*, *Sox11*) and *Il33*, suggesting a role in tissue regeneration [23].

Collectively, these studies highlight the remarkable contextual diversity of Siglec-F^+^ neutrophils within the same anatomical region. They act as APC-like surveillance cells in homeostasis [38], antimicrobial effectors during infection [31], potential modulators of allergic inflammation [24], and contributors to tissue repair [23]. Notably, a unifying theme across these varied roles is the absence of a clearly pathogenic function. Instead, the evidence from the nasal cavity consistently points toward homeostatic, protective, or reparative activities, underscoring the nasal cavity as a unique immunological niche where Siglec-F^+^ neutrophils predominantly exert homeostatic and reparative, rather than pathogenic, functions.

### 3.2. Lung: Pathogenic Inflammation and Tissue Destruction

In stark contrast to their protective roles in the nasal cavity, Siglec-F^+^ neutrophils in the lungs consistently promote pathology across multiple disease models. In elastase-induced emphysema, Hong et al. demonstrated that γδ^+^ T cell-derived IL-17A stimulated airway epithelial and stromal cells to secrete G-CSF, promoting the differentiation of lung-specific pathogenic Siglec-F^+^ neutrophils [29]. These cells were exclusively localized within the lung tissue and bronchoalveolar lavage fluid, suggesting tissue-restricted development rather than bone marrow origin. Functionally, Siglec-F^+^ neutrophils exhibited increased phagocytic activity, enhanced NET formation, and elevated production of TNF-α, IL-6, and IL-1β, while secreting reduced levels of IL-10 compared with conventional neutrophils [29]. The depletion of Siglec-F^+^ neutrophils or pharmacological blockade of their differentiation pathways significantly alleviated emphysema pathology, as evidenced by reduced mean linear intercept scores [29].

In diesel exhaust particle (DEP)-induced asthma models, Shin et al. identified extracellular ATP as a key factor driving Siglec-F^+^ neutrophil differentiation via P2X1 receptor signaling [25]. These cells spontaneously release NETs and produce high levels of cysteinyl leukotrienes by upregulated Ltc4s expression, exacerbating type 2 and type 3 airway inflammation [25]. Adoptive transfer into HDM-induced asthmatic mice markedly increases airway hyperresponsiveness, eosinophil recruitment, and IL-5/IL-13 production by CD4^+^ T cells and ILC2s [25]. This pathogenic role was further confirmed by the therapeutic benefit of combined NET formation blockade (GSK484) and cysteinyl leukotriene receptor antagonism (montelukast) [25], highlighting the potential of Siglec-F^+^ neutrophils as a tractable target in pollution-related asthma.

Notably, Siglec-F^+^ neutrophils have also been observed in a cystic fibrosis-like mouse model. In *Scnn1b-Tg* mice, which overexpress the epithelial sodium channel (ENaC) and exhibit cystic fibrosis-like mucus obstruction and inflammation, Siglec-F^+^ neutrophils were present at baseline in bronchoalveolar lavage and were further recruited during chronic Pseudomonas aeruginosa infection [33]. While the functional role of these cells remained unclear—the authors could not determine whether their recruitment was deleterious or represented a host attempt to clear infection—their presence correlated with exacerbated type 2 and type 3 inflammatory responses, elevated IL-17A levels, and increased tissue damage [33]. This finding extends the pathogenic role of Siglec-F^+^ neutrophils to additional lung disease contexts beyond emphysema and asthma.

More recently, Yu et al. uncovered a distinct mechanism by which pathogenic Siglec-F^+^ neutrophils arise in allergic airway inflammation [27]. Using an eosinophil-specific GPR43 knockout model (*Epx^Cre/+^Gpr43^fl/fl^*), the authors demonstrated that loss of GPR43 signaling in eosinophils led to their hyperactivation and excessive production of IL-4 and GM-CSF. These cytokines directly converted conventional Siglec-F^−^ neutrophils into Siglec-F^+^ subsets characterized by co-expression of PECAM-1, increased cytokine activity (*Il1a*, *Il23a*, *Tnf*), and enhanced ability to promote Th17 differentiation. The accumulation of Siglec-F^+^ neutrophils in the lungs and bronchoalveolar lavage fluid correlated with severe airway inflammation, mucus hypersecretion, and airway hyperresponsiveness. Mechanistically, GPR43 normally functions as a metabolic checkpoint that limits eosinophil overactivation in response to short-chain fatty acids, thereby preventing pathological eosinophil–neutrophil crosstalk. Its deficiency disrupts this restraint, establishing a feed-forward loop between Siglec-F^+^ neutrophils and Th17 cells that exacerbates allergic asthma. Collectively, these findings highlight that impaired eosinophil–neutrophil regulation, through metabolic inactivation of GPR43, represents another pathway by which Siglec-F^+^ neutrophils drive pathogenic lung inflammation.

### 3.3. Cardiovascular System: Consistent Late-Phase Accumulation and Fibrosis

Three independent studies on myocardial infarction revealed remarkably consistent patterns of Siglec-F^+^ neutrophil dynamics in cardiac tissue, with peak accumulation at days 3–4 post-injury and an association with fibrotic remodeling.

Vafadarnejad et al. identified a time-dependent emergence of Siglec-F^+^ neutrophils in a murine model of myocardial infarction (MI) induced by permanent ligation of the left anterior descending coronary artery [34]. Using single-cell RNA sequencing combined with CITE-seq, the authors demonstrated that circulating Siglec-F^−^ neutrophils infiltrate the ischemic myocardium and locally acquire the Siglec-F^+^ phenotype through a process of tissue specification, rather than being preformed in the bone marrow or spleen. These Siglec-F^+^ neutrophils were found exclusively in the infarcted cardiac tissue, constituting over 50% of total Ly6G^+^ neutrophils by day 3 post-MI, whereas they were virtually absent in control, sham-operated, or remote nonischemic hearts. Functionally, they exhibited enhanced phagocytosis of Escherichia coli-derived particles and greater reactive oxygen species (ROS) production than Siglec-F^−^ counterparts. Phenotypically, they showed an aged or activated signature (ICAM1^high^ CD49d^high^ CXCR4^high^ CD62L^low^) and were enriched for Siglecf, Tnf, and Icam1 transcripts, indicating a metabolically active and pro-inflammatory state. However, the study did not identify the molecular factors driving Siglec-F induction, and the authors did not classify these cells as either protective or pathogenic within the context of cardiac injury.

Similarly, Calcagno et al. reported that Siglec-F^+^ neutrophils partially originated in the bone marrow during granulopoiesis but required the ischemic cardiac environment to acquire the full phenotype, including surface Siglec-F protein [35]. Using single-cell transcriptomic profiling, they demonstrated that these cells exhibit strong transcriptional enrichment for Myc signaling, NF-κB activation, and oxidative phosphorylation, consistent with a metabolically reprogrammed, pro-inflammatory state. Notably, by day 4 post-infarction, Siglec-F^+^ neutrophils contributed more than 50% of total NF-κB-regulated gene expression within the cardiac neutrophil compartment [35].

Wang et al. provided mechanistic insights into the pathogenic role of Siglec-F^+^ neutrophils in periodontitis-related myocardial fibrosis [36]. Using both human cohorts and mouse models combining chronic periodontitis (PD) and myocardial infarction (MI), the study demonstrated that persistent PD impaired post-MI recovery through the expansion of scar-associated Siglec-F^+^ neutrophils. Bone marrow neutrophils from PD mice were intrinsically skewed toward this phenotype and differentiated into Siglec-F^+^ cells when exposed to GM-CSF or TGF-β, either individually or synergistically. These cells appeared exclusively in infarcted myocardium—absent in bone marrow, blood, or spleen—indicating a context-dependent, locally reinforced conversion. Functionally, Siglec-F^+^ neutrophils were long-lived and apoptosis-resistant, directly depositing collagen I and fibronectin and activating cardiac fibroblasts via TNFα secretion, thereby promoting excessive extracellular matrix accumulation. PPARγ was identified as the key transcriptional regulator of this differentiation process, and its inhibition markedly reduced Siglec-F^+^ neutrophil abundance and improved cardiac function. Collectively, these findings established Siglec-F^+^ neutrophils as a pathogenic subset that links chronic periodontal inflammation to maladaptive cardiac fibrosis and adverse remodeling following MI.

### 3.4. Kidney: Profibrotic Conversion and Collagen-Secretion

Ryu et al. demonstrated that Siglec-F^+^ neutrophils are essential for creating a profibrotic microenvironment in renal fibrosis models [37]. Using unilateral ureteral obstruction (UUO), adriamycin-induced nephropathy, and ischemia/reperfusion injury models, Siglec-F^+^ neutrophils were found to originate from conventional neutrophils within the renal vasculature rather than the bone marrow. GM-CSF and TGF-β1, produced by both T cells and tubular epithelial cells, induce Siglec-F expression in vitro, with GM-CSF showing a stronger effect [37]. These cells directly produced collagen 1 (COL1A1), making them a dominant immune source of collagen in fibrotic kidneys, while also activating fibroblasts through secretion of TGF-β1, TNF-α, and IL-1β [37]. Their depletion reduced fibrosis more effectively than anti-Ly6G treatment, possibly because Ly6G depletion potentially increases profibrotic eosinophils [37].

### 3.5. Central Nervous System: Th17-Driven Autoimmune

In experimental autoimmune encephalomyelitis (EAE), Hu et al. identified Siglec-F^+^ neutrophils as exacerbators of Th17-mediated neuroinflammation [28]. GM-CSF from pathogenic Th17 cells, alone or synergistically with TGF-β, induced Siglec-F expression on bone marrow neutrophils. These cells exhibited a robust Th17-promoting cytokine profile (IL-23, IL-6, pro-IL-1β, and TNF-α), increased ROS production and NET formation, and promoted Th17 differentiation while suppressing Foxp3^+^ Treg development in coculture experiments [28]. Anti-Siglec-F antibody treatment reduced disease severity in both active and passive EAE models, decreased pathogenic Th17 cells, and increased Tregs without affecting the overall neutrophil population [28].

### 3.6. Spleen: Immunosuppressive Accumulation and Systemic Tolerance in Sepsis

Uniquely among all disease models studied, Liao et al. identified specific accumulation of Siglec-F^+^ neutrophils in the spleen during the immunosuppressive phase of sepsis [32]. This splenic accumulation stands in stark contrast to other infection and inflammatory models: in B. pertussis infection, the spleen shows expansion of conventional Siglec-F- neutrophils rather than Siglec-F^+^ subsets [31]; in periodontitis-related myocardial infarction, Siglec-F^+^ neutrophils are absent from the spleen entirely, appearing only in infarcted myocardium [36]; and in autoimmune EAE, no splenic Siglec-F^+^ neutrophil accumulation occurs [28]. The unique splenic tropism in sepsis likely reflects the spleen’s critical role as a blood-filtering organ during systemic infection, with its specialized architecture of red and white pulp facilitating prolonged retention of these immunosuppressive cells during the transition from hyperinflammation to immunosuppression in persistent inflammation, immunosuppression, and catabolism syndrome (PICS) [32].

In a cecal ligation and puncture (CLP) model, Siglec-F^+^ neutrophils emerged in the spleen from day 3 post-CLP, coinciding with decreased T cell activity [32]. These cells were major IL-10 producers that suppressed CD4^+^ and CD8^+^ T cell activation and cytokine production, and their adoptive transfer into healthy mice reproduced immunosuppression [32]. Importantly, anti-Siglec-F antibody treatment restored T cell function, improved survival after secondary infection, and reduced the bacterial load during E. coli challenge [32]. Recruitment was mediated by T cells and macrophage-derived CXCL1/CXCL2 acting through CXCR2, with inhibition by SB225002 restoring immune function [32].

### 3.7. Common Mechanistic Themes and Tissue-Specific Variations

Despite organ-specific differences, several unifying themes have emerged for noncancerous diseases. First, local conversion from conventional neutrophils is nearly universal, with Siglec-F expression acquired within the target tissues rather than in the bone marrow or circulation [15]. The sole exception is periodontitis-related cardiac fibrosis in which bone marrow priming precedes tissue conversion [8]. Secondly, GM-CSF and G-CSF represent the dominant differentiation signals, although their relative importance varies by organ [15]. Third, IL-17A frequently acts as an indirect orchestrator, promoting cytokine production by non-immune cells, rather than directly inducing Siglec-F expression [29,31]. Fourth, alternative pathways, including ATP/purinergic signaling (lung) and tissue-specific lipid metabolism (liver), can drive differentiation in specific contexts [19,25]. Interestingly, the cystic fibrosis-like airway environment itself may be permissive for Siglec-F^+^ neutrophil emergence, as these cells were detectable even at baseline in Scnn1b-Tg mice with epithelial sodium channel overexpression-induced mucus obstruction, independent of specific differentiation factors [33].

The functional dichotomy between protective and pathogenic roles appears to be determined by the balance between antimicrobial function and tissue-damaging inflammation. In the nasal cavity, where rapid pathogen clearance is paramount and the tissue architecture is relatively simple, Siglec-F^+^ neutrophils provide beneficial antimicrobial activity. By contrast, in the delicate alveolar structures of the lung or functionally critical myocardium, enhanced inflammatory capacity and prolonged survival lead to pathological tissue remodeling and organ dysfunction.

## 4. Siglec-F^+^ Neutrophils in Cancer: The Paradox of Pro-Tumor Default and Immunotherapy-Induced Reprogramming

### 4.1. The Pro-Tumor Default: A Stable and Dominant Paradigm

Within the tumor microenvironment (TME), Siglec-F^+^ neutrophils predominantly exhibit pro-tumorigenic functions that facilitate cancer progression. This pro-tumor paradigm, characterized by potent immunosuppression and direct tumor support, has been most extensively elucidated in lung adenocarcinoma.

**Figure 2:**
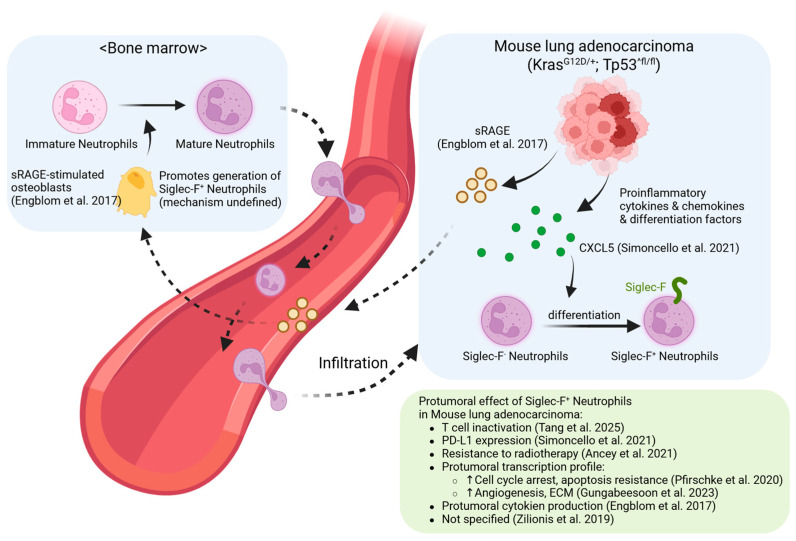
Generation and Functions of Siglec-F^+^ Neutrophils in Cancer: Siglec-F^+^ neutrophils are supplied from the bone marrow or differentiate locally within the tumor microenvironment (TME), acquiring diverse pro-tumorigenic functions. Potent immunotherapy can, however, reprogram these cells into anti-tumor cytotoxic effectors. Upward (↑) and downward (↓) arrows denote an increase and a decrease in phenotype or ex-pression, respectively [11,12,13,14,15,16].

Of various tumor models, lung adenocarcinoma model (*Kras*^G12D/+^; *Tp53*^fl/fl^, commonly referred to as the KP model) stands out as the most extensively investigated [11,12,13,14,15,16]. Tumors arising in this model collectively display distinct pro-tumoral characteristics. The integrated mechanisms underlying these features are detailed in Figure 2.

Osteoblasts in the bone marrow remotely supply lung tumors with these neutrophils [11]. Mechanistically, elevated serum sRAGE in tumor-bearing mice promoted CXCR2 expression on developing neutrophils, facilitating their egress from the bone marrow. The essential role of osteoblasts was confirmed through parabiosis experiments, where their depletion reduced both neutrophil counts and tumor growth [11].

Further characterization in lung adenocarcinoma models highlighted the remarkable longevity of these cells, with a half-life of 3–4 days, and their tight correlation with tumor burden [13,14]. Functionally, they promoted tumor growth by enhancing ROS production, supporting macrophage differentiation, and directly stimulating tumor cell proliferation. This population was also found to correspond to a human hN5 neutrophil subset associated with poor patient survival [13]. The chemokine CXCL5 was identified as another critical factor for their accumulation; its deletion in tumor cells markedly reduced their infiltration. These neutrophils were shown to express high levels of PD-L1, actively suppressing local CD8^+^ T cell responses and limiting the efficacy of anti-PD-L1 antibody therapy [15].

This pro-tumorigenic role extends beyond lung cancer. In hepatocellular carcinoma (HCC) arising from metabolic dysfunction-associated steatohepatitis (MASH), these neutrophils are generated through the synergistic action of GM-CSF and linoleic acid [19]. They promote tumor cell stemness and proliferation via TGF-β and suppress immune recognition by reducing tumor cell MHC-I expression [19]. Similarly, in head and neck cancers, tumor-derived G-CSF and GM-CSF promote the survival of aged, immunosuppressive neutrophils equipped with an arsenal of inhibitory molecules (PD-L1, VISTA, Arg1, ROS), which effectively suppress CD8^+^ T cell function and accelerate their exhaustion [21].

Further defining their potent suppressive identity, studies have linked Siglec-F expression directly to the most aggressive subset of polymorphonuclear myeloid-derived suppressor cells (PMN-MDSCs). Veglia et al. identified three distinct tumor-infiltrating neutrophil populations, with the most immunosuppressive subset (termed PMN3) being characterized by high co-expression of CD14 and Siglec-F [18]. These CD14^high^ Siglec-F^+^ cells exhibited the highest levels of suppressive molecules like Arg1 and PD-L1 and were most effective at inhibiting T cell function. Their activation occurred locally within the tumor, driven by factors such as GM-CSF and hypoxia. Importantly, the gene signature of these cells strongly correlates with poor clinical outcomes in cancer patients, cementing their role as a key barrier to anti-tumor immunity [18].

### 4.2. Modulation During Therapy: From Cytokine Paradoxes to Immunotherapy-Induced Reprogramming

Modern cancer therapy, often combining chemotherapy and immunotherapy, significantly alters the tumor microenvironment (TME). Within this altered context, Siglec-F^+^ neutrophils exhibit remarkable functional plasticity, shifting between immunosuppressive and anti-tumor activities depending on the therapeutic intervention. A key example of this functional conversion was reported in a preclinical melanoma model by Hirschhorn et al., who utilized an aggressive triple-combination therapy consisting of cyclophosphamide preconditioning, adoptive transfer of Trp1-specific CD4^+^ T cells, and anti-OX40 co-stimulation [17]. This regimen successfully reprogrammed the function of Siglec-F^+^ neutrophils from a pro-tumorigenic to an anti-tumorigenic state. While the activated T cells mediated antigen-specific killing, the reprogrammed neutrophils induced iNOS-dependent bystander cytotoxicity. This neutrophil-mediated activity was crucial for eliminating antigen-loss tumor variants that would otherwise evade T-cell recognition, leading to complete tumor eradication.

These therapy-activated Siglec-F^+^ neutrophils exhibited a distinct molecular profile. Surface marker analysis revealed upregulation of activation-associated molecules (CD14, CD117, IL-1Rβ, CD18, CD40, CD177, CD49d) and downregulation of suppressive markers (CD62L, CD206, PD-L1). Single-cell RNA sequencing identified unique transcriptional programs enriched for genes involved in activation, chemotaxis, and nitric oxide biosynthesis, including *Il1b*, *Osm*, *Plaur*, *Socs3*, *Ccl4*, and *G0s2*.

Functionally, these reprogrammed neutrophils demonstrated enhanced NET formation and iNOS-mediated cytotoxicity. The therapeutic intervention reshaped the TME with elevated levels of neutrophil-activating cytokines and chemokines, including GM-CSF, IL-3, MIP-1α, and MIP-1β. Depletion studies using anti-Ly6G antibodies confirmed their essential role, as treatment efficacy was abolished in their absence. Importantly, the anti-tumor neutrophil gene signature correlated with improved survival in melanoma patients receiving anti-PD-1 therapy, suggesting clinical relevance [17].

However, successful reprogramming of Siglec-F^+^ neutrophils appears to be an exception rather than the norm. These divergent outcomes likely reflect variations in tumor types and therapeutic regimens. While Hirschhorn et al.‘s aggressive triple-combination therapy achieved reprogramming [40], Gungabeesoon et al. found that conventional immunotherapies (anti-CD40, anti-PD-1) or standard chemotherapy failed to alter Siglec-F^+^ neutrophil populations in lung and colon carcinoma models [16]. This suggests that reprogramming pro-tumor neutrophils requires exceptionally intensive therapeutic combinations that generate a highly inflammatory TME. The stability of the Siglec-F^+^ pro-tumor state suggests that effective therapies may achieve tumor control through alternative mechanisms, such as expanding distinct anti-tumor neutrophil subsets rather than converting existing populations.

## 5. Therapeutic Implications of Siglec-F^+^ Neutrophil Biology

The context-dependent nature of Siglec-F^+^ neutrophils necessitates distinct therapeutic strategies: enhancing their beneficial functions in protective contexts while suppressing their detrimental effects in pathological settings.

### 5.1. Therapeutic Strategies in Non-Cancer Contexts

In protective settings, therapies should aim to enhance the beneficial functions of Siglec-F^+^ neutrophils. During nasal infections like B. pertussis, these cells are crucial for mediating bacterial clearance through enhanced NET formation and antimicrobial peptide production [31]. In such cases, strategies could include targeted delivery of GM-CSF or IL-17A to boost their antimicrobial activity. Their extended lifespan (3–4 days) also makes them ideal vehicles for sustained therapeutic delivery using neutrophil membrane-coated nanoparticles, which can leverage their innate tissue-infiltrating properties for targeted drug delivery to infection sites [47,48].

Conversely, in pathogenic inflammatory diseases, suppressing their function is key. This can be achieved by exploiting cellular antagonism between eosinophils and neutrophils. As demonstrated by Yu et al., eosinophil-specific loss of GPR43 leads to their hyperactivation and excessive secretion of IL-4 and GM-CSF, which converts conventional neutrophils into pathogenic Siglec-F^+^ subsets. These neutrophils then up-regulate PECAM-1, enabling direct adhesion to eosinophils and establishing a self-reinforcing inflammatory circuit. A therapeutic strategy could therefore involve a dual-axis approach: (1) interrupting this pro-inflammatory cascade by blocking IL-4, GM-CSF, and PECAM-1, and (2) reinforcing the metabolic checkpoint maintained by SCFA–GPR43 signaling [27].

### 5.2. Therapeutic Strategies in Cancer

In cancer, where Siglec-F^+^ neutrophils are predominantly pathogenic, multiple intervention points are available.

#### 5.2.1. The Choice Between Depletion and Reprogramming

A significant therapeutic opportunity lies in the functional reprogramming of these cells. As demonstrated in a melanoma model, an aggressive triple-combination therapy (cyclophosphamide, adoptive CD4^+^ T cells, and anti-OX40) successfully converted these cells from a pro-tumorigenic to an anti-tumorigenic state [17]. These reprogrammed cells upregulated activation markers (CD14, CD117) and downregulated suppressive molecules (PD-L1, CD206), driven by a TME rich in GM-CSF, IL-3, and MIP-1α/β, which enabled them to perform bystander killing of antigen-loss tumor variants.

When reprogramming is not feasible, direct depletion is a viable alternative. This can be achieved using anti-Siglec-F or anti-Ly6G antibodies, which effectively reduce tumor burden and sensitize tumors to immunotherapy [19,37]. Furthermore, the sialic acid-binding properties of Siglec-F provide opportunities for highly targeted elimination using synthetic ligands that mimic structures like 6′-sulfo-sialyl Lewis X [49].

#### 5.2.2. Targeting Metabolic and Functional Vulnerabilities

Prior studies focusing on the unique metabolic state of pro-tumoral neutrophils provides valuable insights. Within hypoxic tumors, HIF-1α activation drives a glycolytic switch that supports their immunosuppressive functions [50]. Their enhanced GLUT1 expression allows them to outcompete T cells for glucose, creating metabolic “nutrient deserts” that impair anti-tumor immunity [14,51]. Arginase-1 is another important target; its inhibition with CB-1158 restored T-cell responses in clinical trials by increasing local arginine availability [52]. Additionally, targeting survival pathways like PI3K-AKT could selectively induce apoptosis in these cells [18].

#### 5.2.3. Overcoming Immunotherapy Resistance

Siglec-F^+^ neutrophils are a notable contributor to resistance against checkpoint blockade. A preventive strategy is to block their generation and recruitment by targeting upstream signals like GM-CSF [13,27,36], G-CSF [29], or CXCL5 [37]. More specific pathways that can be targeted include the linoleic acid–c-Myc axis in MASH-HCC and the IL-6–STAT3 signaling driven by cancer-associated fibroblasts [19]. CXCR2 inhibition, in particular, has shown promise in suppressing metastasis and augmenting immunotherapy [53].

Once present in the TME, these neutrophils act as “therapeutic sinks” that sequester anti-PD-L1 antibodies; mathematical modeling confirms that therapeutic responses are more sensitive to PD-L1 levels on immune cells than on tumor cells [44,45,54]. They also directly induce T cell exhaustion via their own PD-L1 expression [21,51]. Therefore, combination strategies that deplete these neutrophils before or during checkpoint blockade may be required to overcome this multifaceted resistance.

## 6. Future Directions: Bridging the Translational Gap

A critical gap remains in translating murine Siglec-F^+^ neutrophil findings to human disease. Overcoming this challenge requires addressing fundamental biological questions while simultaneously establishing human relevance.

### 6.1. Fundamental Questions in Siglec-F^+^ Neutrophil Biology

Despite phenotypic characterization, core biological mechanisms remain unresolved. The molecular mechanisms governing Siglec-F^+^ neutrophil differentiation require systematic multi-omics analyses—integrating single-cell RNA sequencing with chromatin accessibility (scATAC-seq), proteomics (CITE-seq), and metabolomics. These approaches will identify the core transcriptional, epigenetic, and metabolic programs that distinguish protective from pathogenic Siglec-F^+^ states and reveal the specific signals driving their context-dependent functions. The acquisition of an eosinophil-associated marker (Siglec-F) by neutrophils raises fundamental questions about cellular origin and heterogeneity. Inducible fate-mapping and time-stamping models, similar to those used to dissect macrophage heterogeneity [55], can definitively determine whether these cells arise from conventional neutrophils via reprogramming or represent a distinct developmental lineage. Combining these approaches with spatial transcriptomics will clarify whether protective and pathogenic populations represent different lineages or distinct activation states of a common precursor.

### 6.2. The Critical Translational Gap: Human Siglec-8^+^ Neutrophils

Translating findings from murine Siglec-F^+^ neutrophils to human pathology represents the most critical challenge. While Siglec-8, the human paralog of murine Siglec-F, is well-characterized on eosinophils, mast cells, and basophils [56], its presence on neutrophils has been largely unexplored. To date, only a single pioneering study has reported Siglec-8^+^ neutrophils in human disease [37].

That investigation into renal fibrosis provided the first evidence for a human counterpart. Analysis of the Nephroseq database revealed that Siglec-8 and neutrophil-marker expression were elevated in diseased kidney tissues and correlated with disease severity. Flow cytometry detected Siglec-8^+^ neutrophils in fibrotic regions of human kidney tumors, and human neutrophils could be induced to express Siglec-8 in vitro with GM-CSF and TGF-β1 [37]. However, the investigation was limited by an inability to isolate and functionally characterize these cells directly from patient tissue.

While these initial findings are promising, they underscore an urgent need for comprehensive validation across multiple human diseases. Until systematic validation is accomplished, the significant therapeutic potential of targeting this neutrophil subset remains speculative.

### 6.3. Roadmap for Clinical Translation

Multi-omics validation [5]: Single-cell profiling of patient-derived neutrophils across cancer, fibrosis, and inflammatory diseases must determine if human Siglec-8^+^ populations share defining features of murine counterparts—extended lifespan, metabolic reprogramming, and context-dependent functions.

Model development: Humanized mouse models expressing human Siglec-8 are essential for testing targeted therapeutics. These models must recapitulate the tissue-specific generation and functional diversity observed in murine systems.

Biomarker identification: Development of real-time biomarkers to assess neutrophil state and plasticity in patients will enable precision medicine approaches. This includes identifying surface markers, metabolic signatures, and functional readouts that predict therapeutic responsiveness.

Clinical study design: Future trials must consider the context-dependent nature of these cells. Therapeutic strategies that deplete pathogenic populations in cancer may need modification for inflammatory diseases where the same cells might have protective roles. Temporal considerations—when to intervene based on disease stage—will be crucial for optimizing outcomes.

Only through this comprehensive approach can we harness the therapeutic potential suggested by murine studies and develop effective treatments targeting this sophisticated neutrophil subset in human disease.

## 7. Conclusions

Siglec-F^+^ neutrophils represent a fundamental revision of neutrophil biology. They are not short-lived, homogeneous cells but rather a heterogeneous population defined by remarkable plasticity, with extended lifespans, sophisticated metabolic programs, and context-dependent functions. Their phenotype is acquired through tissue-specific reprogramming, primarily driven by GM-CSF in combination with local factors such as TGF-β, linoleic acid, or extracellular ATP. This enables sustained and powerful effects on the microenvironment that vary dramatically by anatomical location and disease context.

The functional dichotomy of Siglec-F^+^ neutrophils exemplifies their plasticity. In the nasal mucosa, they provide essential antimicrobial defense through enhanced NET formation and bacterial clearance. Yet in the lungs, the same effector functions drive pathological inflammation and tissue destruction. They orchestrate fibrotic remodeling in heart and kidneys through direct collagen deposition, while in sepsis, they accumulate specifically in the spleen to induce systemic immunosuppression. In cancer, they emerge as master regulators of immune evasion, deploying metabolic warfare through arginase-1 expression and creating immunosuppressive niches via PD-L1. The discovery that osteoblasts can remotely supply tumors with these cells reveals unexpected systemic regulation of their generation.

Critically, the paradoxical pro-survival effect of Siglec-F in neutrophils—contrasting with its pro-apoptotic role in eosinophils—highlights fundamental differences in cell type-specific signaling that remain incompletely understood. Their relationship to existing neutrophil classifications (N1/N2, PMN-MDSCs, HDNs/LDNs) suggests they may represent a terminally differentiated state arising from multiple pathways rather than a distinct lineage, though this requires definitive investigation through lineage tracing studies.

The therapeutic implications are equally complex. While selective depletion, differentiation blockade, and metabolic targeting all show promise, the most striking finding is that potent combination immunotherapy can reprogram pro-tumor Siglec-F^+^ neutrophils into tumor-killing effectors. This functional conversion, rather than simple elimination, may represent the optimal therapeutic strategy—but only when timing and context are carefully considered, as premature depletion can impair beneficial tissue repair.

Despite this progress, the therapeutic potential remains unrealized due to a critical translational gap. Human Siglec-8^+^ neutrophils are virtually unstudied, with only a single report in renal fibrosis. It remains unknown whether they represent true functional equivalents of murine Siglec-F^+^ cells or if human neutrophils achieve similar functions through different molecular programs. Moving forward, high-resolution single-cell characterization across species, time, and space is essential. Such studies must identify conserved functional states—whether Siglec-8^+^ or otherwise—and reveal the molecular switches governing protective versus pathogenic fate decisions. Only through this systematic dissection can we develop next-generation immunotherapies that precisely target or reprogram these sophisticated cellular chameleons to benefit patients across the spectrum of inflammatory, fibrotic, and neoplastic diseases.

## Figures and Tables

**Table 1:** Organ-specific manifestations of Siglec-F^+^ neutrophils in non-cancerous diseases. Upward (↑) and downward (↓) arrows denote an increase and a decrease in phenotype or ex-pression, respectively.

Location	Context	Key Drivers	Key Features	Outcome	References
Nasal cavity	Steady-State	Not identified	Long-lived; Hyper-segmented nuclei with dendritic processes; APC-like cross-presentations to CD8^+^ T cells	Homeostatic	Gonzalez et al. (2024) [38]
	*B. pertussis* Infection	IL-17A	Tissue-resident; Enhanced NETosis; Produce antimicrobial peptides (S100A8, LCN2).	Protective	Borkner et al. (2021) [31]
	OVA-induced Rhinitis	Ovalbumin	“Botryoid” nuclei; Enhanced phagocytosis and ROS production.	Unclear	Matsui et al. (2020) [24]
	Olfactory Neuroepithelium	LPS (for conversion); MMZ (for accumulation)	Bone marrow-derived, local conversion; Retain NETosis capacity; Express neurogenesis-related genes (*Efna5*, *Sox11*, *Il33*).	Reparative	Ogawa et al. (2021) [23]
Lung	PPE-induced Emphysema	G-CSF secreted by stromal cells, which is induced by IL-17A from γδ^+^ T cells	Pro-inflammatory cytokine profile (↑TNF-α, IL-6, IL-1β; ↓IL-10); Enhanced NETosis and phagocytosis.	Pathogenic	Hong et al. (2024) [29]
	DEP-induced Asthma	Extracellular ATP (via P2X1 receptor)	Spontaneous NET release; High cysteinyl leuko-triene production (via *Ltc4s*).	Pathogenic	Shin et al. [25]
	Cystic Fibrosis-like Disease (Scnn1b-Tg mice)	Chronic P. aeruginosa infection/Mucus obstruction	Presence correlates with exacerbated inflammation and tissue damage.	Unclear	Vaillancourt et al. [33]
	Allergic Airway Inflammation	Eosinophil-derived IL-4 and GM-CSF	PECAM-1 co-expression; Enhanced Th17-promoting cytokine activity (*Il1a*, *Il23a*, *Tnf*).	Pathogenic	Yu et al. (2024) [27]
Heart	Myocardial Infarction (MI)	Not identified	Pro-inflammatory transcriptome (TNF, Myc, NF-κB, OxPhos) [35]; Aged/activated phenotype; Enhanced phagocytosis and ROS [34].	Not classified (pro-inflammatory)	Calcagno et al. (2021) [35], Vafadarnejad et al. (2020) [34]
	Periodontitis-related MI	GM-CSF/TGF-β on predisposed bone marrow neutrophils; PPARγ	Long-lived, apoptosis-resistant; Directly deposit Collagen I and Fibronectin; Activate fibroblasts via TNFα.	Pathogenic	Wang et al. (2025) [36]
Kidney	Fibrosis (UUO, ADR, I/R)	T cells and Tubular epithelial cells (GM-CSF and TGF-β1)	Directly produce Collagen I (COL1A1); Activate fibroblasts (TGF-β1, TNF-α, IL-1β).	Pathogenic (profibrotic)	Ryu et al. (2022) [37]

## Data Availability

No new data were created or analyzed in this study.

## Abbreviations

The following abbreviations are used in this manuscript:

PMN	Polymorphonuclear neutrophil
Siglec-F	Sialic acid-binding immunoglobulin-like lectin F
TAN	Tumor-associated neutrophil
HCC	Hepatocellular carcinoma
G-CSF	Granulocyte colony-stimulating factor
NET	Neutrophil extracellular trap
PMN-MDSC	Polymorphonuclear myeloid-derived suppressor cell
BALF	Bronchoalveolar lavage fluid
LDN	Low-density neutrophil
HDN	High-density neutrophil
ITIM	Immunoreceptor tyrosine-based inhibitory motif
ROS	Reactive oxygen species
DEP	Diesel exhaust particle
UUO	Unilateral ureteral obstruction
EAE	Experimental autoimmune encephalomyelitis
CLP	Cecal ligation and puncture
PICS	Persistent inflammation, immunosuppression, and catabolism syndrome
sRAGE	Soluble receptor for advanced glycation end-products
MASH	Metabolic dysfunction-associated steatohepatitis
